# A Genetic Association Study of Serum Acute-Phase C-Reactive Protein Levels in Rheumatoid Arthritis: Implications for Clinical Interpretation

**DOI:** 10.1371/journal.pmed.1000341

**Published:** 2010-09-21

**Authors:** Benjamin Rhodes, Marilyn E. Merriman, Andrew Harrison, Michael J. Nissen, Malcolm Smith, Lisa Stamp, Sophia Steer, Tony R. Merriman, Timothy J. Vyse

**Affiliations:** 1 Rheumatology Section, Division of Medicine, Imperial College London, London, United Kingdom; 2 Department of Biochemistry, University of Otago, Dunedin, New Zealand; 3 Department of Medicine, Wellington School of Medicine and Health Sciences, University of Otago, Dunedin, New Zealand; 4 Rheumatology Unit, Repatriation General Hospital, Daw Park, Adelaide, South Australia, Australia; 5 Department of Medicine, University of Otago, Christchurch, New Zealand; 6 Clinical and Academic Rheumatology, Kings College Hospital NHS Foundation Trust, London, United Kingdom; University of London, United Kingdom

## Abstract

**Background:**

The acute-phase increase in serum C-reactive protein (CRP) is used to diagnose and monitor infectious and inflammatory diseases. Little is known about the influence of genetics on acute-phase CRP, particularly in patients with chronic inflammation.

**Methods and Findings:**

We studied two independent sets of patients with chronic inflammation due to rheumatoid arthritis (total 695 patients). A tagSNP approach captured common variation at the *CRP* locus and the relationship between genotype and serum CRP was explored by linear modelling. Erythrocyte sedimentation rate (ESR) was incorporated as an independent marker of inflammation to adjust for the varying levels of inflammatory disease activity between patients. Common genetic variants at the *CRP* locus were associated with acute-phase serum CRP (for the most associated haplotype: *p* = 0.002, *p<*0.0005, *p<*0.0005 in patient sets 1, 2, and the combined sets, respectively), translating into an approximately 3.5-fold change in expected serum CRP concentrations between carriers of two common *CRP* haplotypes. For example, when ESR = 50 mm/h the expected geometric mean CRP (95% confidence interval) concentration was 43.1 mg/l (32.1–50.0) for haplotype 1 and 14.2 mg/l (9.5–23.2) for haplotype 4.

**Conclusions:**

Our findings raise questions about the interpretation of acute-phase serum CRP. In particular, failure to take into account the potential for genetic effects may result in the inappropriate reassurance or suboptimal treatment of patients simply because they carry low-CRP–associated genetic variants. CRP is increasingly being incorporated into clinical algorithms to compare disease activity between patients and to predict future clinical events: our findings impact on the use of these algorithms. For example, where access to effective, but expensive, biological therapies in rheumatoid arthritis is rationed on the basis of a DAS28-CRP clinical activity score, then two patients with identical underlying disease severity could be given, or denied, treatment on the basis of *CRP* genotype alone. The accuracy and utility of these algorithms might be improved by using a genetically adjusted CRP measurement.

*Please see later in the article for the Editors' Summary*

## Abstract 2

A genetic association study by Timothy Vyse and colleagues suggests that there is a significant association between CRP variants and acute-phase serum CRP concentrations in patients with rheumatoid arthritis, including those with chronic inflammation.

## Editors' Summary

C-reactive protein (CRP) is a serum marker for inflammation or infection and acts by binding to a chemical (phosphocholine) found on the surface of dead or dying cells (and some types of bacteria) in order to activate the immune system (via the complement system). Fat cells release factors that stimulate the liver to produce CRP, and serum levels greater than 10 mg/l are generally considered indicative of an infectious or inflammatory process. After an inflammatory stimulus, serum CRP levels may exceed 500 times baseline, so CRP is used in all medical specialities to help diagnose inflammation and infection. Although patients with chronic inflammatory diseases, such as rheumatoid arthritis, have raised levels of CRP, levels of CRP are still highly variable. Some studies have suggested that there may be genetic variations of CRP (CRP variants) that determine the magnitude of the acute-phase CRP response, a finding that has important clinical implications: CRP thresholds are used as a diagnostic component of formal clinical algorithms and play an important role in a clinician's decision-making process when diagnosing inflammatory disease and choosing treatment options. Therefore, it is possible that false reassurance could be given to a patient with disease, or optimal treatment withheld, because some patients are genetically predisposed to have only a modest increase in acute-phase CRP.

**Why Was This Study Done?:**

Although some studies have looked at the CRP gene variant response, few, if any, studies have examined the CRP gene variant response in the context of chronic inflammation, such as in rheumatoid arthritis. Therefore, this study aimed to determine whether CRP gene variants could also influence CRP serum levels in rheumatoid arthritis.

**What Did the Researchers Do and Find?:**

The authors studied two independent sets of patients with chronic inflammation due to rheumatoid arthritis (total 695 patients): one patient set used a cohort of 281 patients in the UK, and the other patient set (used for replication) consisted of 414 patients from New Zealand and Australia. A genetic technique (a tagSNP approach) was used to capture common variations at the *CRP* locus (haplotype association analysis) at both the population and the individual level. The relationship between genotype and serum CRP was explored by linear modeling. The researchers found that common genetic variants at the *CRP* locus were associated with acute-phase serum CRP in both patient sets translating into an approximate 3.5-fold change in expected serum CRP between carriers of two common *CRP* variants. For example, when ESR = 50 mm/h the expected CRP serum level for one common CRP variant was 43.1 mg/l and for another CRP variant was 14.2 mg/l.

**What Do These Findings Mean?:**

The findings of this study raise questions about the interpretation of acute-phase serum CRP, as they suggest that there is a significant association between *CRP* variants and acute-phase serum CRP concentrations in a group of patients with rheumatoid arthritis, including those with chronic active inflammation. The size of the genetic effect may be large enough to have a clinically relevant impact on the assessment of inflammatory disease activity, which in turn may influence therapeutic decision making. Failure to take into account the potential for genetic effects may result in the inappropriate reassurance or undertreatment of patients simply because they carry low-CRP–associated genetic variants. CRP is increasingly being incorporated into clinical algorithms to compare disease activity between patients and to predict future clinical events, so these findings impact on the use of such algorithms. The accuracy and utility of these algorithms might be improved by using a genetically adjusted CRP measurement.

**Additional Information:**

Please access these Web sites via the online version of this summary at http://dx.doi.org/10.1371/journal.pmed.1000341

- Lab Test Online provides information on CRP
- The Wellcome Trust provides a glossary of genetic terms
- Learn.Genetics provides access to the Genetic Science Learning Center, which is part of the human genome project

## Introduction

Genetic variants at the *CRP* locus influence the low, basal levels of serum C-reactive protein (CRP) in apparently healthy individuals [1]–[5]. The primary motivation for these existing studies has been the observation of a strong correlation between basal CRP and future cardiovascular risk, and the possibility, now looking less likely, that CRP has a causal role in this process [6],[7]. The widespread clinical application of CRP measurement, however, is not related to its low-level variation within the basal range, but to the high levels seen as part of the human acute-phase response. Serum CRP levels greater than 10 mg/l are generally considered indicative of an infectious or inflammatory process [8]. CRP expression increases rapidly following an inflammatory stimulus, leading to serum levels that may exceed 500 times baseline, making CRP an ideal marker for the diagnosis and monitoring of inflammatory processes. Serum CRP is extensively used in all medical specialities for this purpose [8]. Surprisingly, there has been little published data on the role of *CRP* variants in determining the magnitude of this acute-phase response, and none in the context of chronic active inflammation.

The demonstration of a strong, genetically determined and hence lifelong, influence on the magnitude of acute-phase CRP in any particular patient would have a number of important implications. Firstly, as a diagnostic test, CRP thresholds are used not only as a component of formal clinical algorithms, but also as part of a physician's intuitive decision-making process when diagnosing inflammatory disease and making treatment decisions. As we discuss later, this raises the possibility that false reassurance could be given to a patient with disease, or optimal treatment withheld, because they are genetically predisposed to only a modest rise in acute-phase CRP. Secondly the demonstration of a strong genetic effect on the acute-phase CRP response also raises the possibility that this may have consequences in terms of individual susceptibility to diseases in which the innate immune system plays a protective (or deleterious) role.

The existing literature on the effects of *CRP* genetics on acute-phase CRP levels consists of five studies, each of which measured the magnitude of CRP rise following a single noxious stimulus [9]–[13]. As we discuss later, this approach may be complicated by the difficulty in quantifying or standardising stimuli between patients or a difficulty in inducing a marked CRP rise. We have therefore taken a different approach, which we believe offers distinct advantages. We have studied genetic determinants of acute-phase CRP in patients with rheumatoid arthritis, a chronic inflammatory disease characterised by a marked acute-phase response.

By studying patients with rheumatoid arthritis we made use of the historical tendency to record the erythrocyte sedimentation rate (ESR) alongside CRP as an alternative and independent marker of inflammation. Although ESR has become less widely used because of its slow response following an inflammatory stimulus, this is not important in chronic inflammation where both CRP and ESR are at a steady state. The factors contributing to ESR are not fully understood, but seem to be related to intrinsic erythrocyte membrane properties, notably the charge, with a lesser influence from the concentration of large plasma proteins such as immunoglobulin or fibrinogen [14],[15]. There is no evidence that serum CRP levels directly influence ESR: indeed the slow fluctuation in ESR following the onset or resolution of inflammation is in stark contrast to the rapid expression and short half-life of CRP [8],[16],[17]. The incorporation of ESR as a covariate in our model fulfils two purposes. First, it provides an independent adjustment for the underlying inflammatory status of each of our patients (a consequence of their synovial joint inflammation). Without this adjustment, the random error associated with dramatically differing levels of inflammation across the cohort (random with respect to either recruitment into the study, or with respect to underlying *CRP* genotype) may be so great as to make the relatively small effect of *CRP* genotype on CRP levels difficult to demonstrate with any level of significance. Second, since most physicians have an intuitive appreciation of the relationship between CRP and ESR in a variety of inflammatory settings, the incorporation of ESR into our models allows us to generate a clinically interpretable estimate of the magnitude of genetic effect at all levels of underlying inflammatory drive. Our results are therefore directly relevant to the conditions in which CRP quantification contributes to clinical decision making.

In this paper we study the association between genetic variants at the *CRP* locus and acute-phase CRP in patients with chronic active inflammation due to rheumatoid arthritis.

## Methods

### Study Participants

The discovery study was carried out in a cohort of 281 UK rheumatoid arthritis cases (patient set 1). Details on the recruitment of these patients have been published elsewhere [18]. The mean age of participants was 61 y (SD 12), 79% were female. Replication was performed in 414 rheumatoid arthritis patients recruited in New Zealand (Wellington and Christchurch) and Australia (Adelaide) (patient set 2). The mean age was 62 y (SD 13) and 70% were female. All patients were of self-reported white European ancestry and met 1987 ACR revised criteria for the diagnosis of rheumatoid arthritis [19]. Data were available for at least one pair of measurements for CRP and ESR (Westergren method) collected simultaneously as part of routine clinical management. For some of the individuals in patient set 2, data were available for serial CRP/ESR pairs, measured at different times. For these individuals, each CRP/ESR pair was arbitrarily assigned an integer value, and a random number generator used to identify one of these pairs at random to carry forward for analysis.

Ethical approval for the collection of DNA and patient data was obtained locally at each recruitment site (from the Guy's hospital Research Ethics Committee, London UK, the University Hospital Lewisham ethics committee, London, UK, the Upper South B regional ethics committee of New Zealand, the MultiRegion Ethics committee of New Zealand, and the Research and Ethics committee, Repatriation General Hospital, Adelaide Australia). Written informed consent was obtained from each patient. Patient identity was encrypted at the site of collection and all data were analysed anonymously.

### SNP Selection and Genotyping

SNPs were selected to tag common variation at *CRP*. We used previously published genotype data on 22 SNPs densely covering *CRP* in 799 unrelated, healthy UK individuals as our reference dataset for this population [20]. Using these data in turn captured all of the *CRP* variation observed in the HapMap CEPH population. Tagging SNPs were selected using the tagger facility of Haploview (employing an aggressive tagging strategy, *r* ^2^ = 0.9 threshold) [21].

Genotyping on patient set 1 was performed by matrix-assisted laser desorption and ionization-time of flight (MALDI-TOF) mass spectrometry (Sequenom) using the iPLEX assay. Genotyping on patient set 2 was outsourced to Kbioscience Ltd using their variant of competitive allele-specific PCR (KASPar) (www.kbioscience.co.uk/chemistry/chemistry_application_note.html). Duplicate genotyping was performed on 5% of patients to ensure reproducibility (100% concordance observed). Predefined quality control exclusion criteria were: any individual genotyped at <90% of markers, any marker genotyping in <90% individuals, any marker with a minor allele frequency <5%, and any marker out of Hardy-Weinberg equilibrium (*p*<0.01). Ten individuals were excluded from patient set 2 because of poor genotyping, otherwise genotyping quality control criteria were met and all markers were in Hardy-Weinberg equilibrium. Population haplotype structure was inferred using Haploview, and individual patient haplotypes were inferred using PHASE v. 2.1 [21],[22]. Only individuals for whom haplotypes could be assigned with a posterior probability >0.95 were carried forward for haplotype association analysis. Six individuals were excluded after failing to meet this requirement.

### Statistical Analysis

The standard CRP assay becomes nonlinear below 1 mg/l leading to the common practice of reporting values as a range (i.e., <1) or rounded to an integer (i.e., “0”). We assigned all CRP measurements reported as <1 mg/l a value of 0.5 mg/l. An initial review identified four data outliers with very low ESR but increased CRP; three from patient set 1 and one from patient set 2. We postulated that these individuals were in the early stages of an acute inflammatory episode (when the rise in ESR lags behind the rise in CRP) and excluded them from further analysis.

Both CRP and ESR distributions were positively skewed so a log-transformation was applied for analysis. The association between SNP genotype and the quantitative markers of inflammation (CRP or ESR) was evaluated by linear model, with genotype modelled as an additive allelic effect. All parameter estimates given throughout the paper are expressed relative to the major allele. An initial assessment was made of the association between *CRP* SNPs and the “raw” serum CRP levels, unadjusted for ESR. In addition, the direct association between *CRP* SNPs and ESR itself was made. In all subsequent models the association between CRP genotype (either at SNP or haplotype level) was assessed with ESR, as an independent marker of background inflammatory status, included as a covariate in the model. The relationship between CRP and ESR was nonlinear, so the final model included a significant quadratic term:



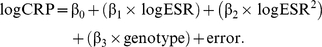



Since patients and data in this study originated from four sites of collection (South London, Wellington, Christchurch, and Adelaide), a random effects parameter was included where relevant to reflect these clusters of data.

In the haplotype analyses, a comparison of haplotype effect was achieved by simultaneously entering each haplotype into the model with the remaining, commonest, haplotype (H1) acting as the reference. Expected CRP values were derived from the regression parameters by back-transforming to the original scale, with the mean of the log-distribution reflecting the median of the untransformed distribution.

## Results

In patient set 1 the median serum CRP was 11 mg/l (range 1–195; 5th, 95th centiles 5, 88). 51.8% of CRP measurements were >10 mg/l. The median ESR was 22.5 mm/h (range 2–132; 5th, 95th centiles 4, 83). The six genotyped SNPs (with minor allele frequency) were rs2808632 (0.28), rs3093059 (0.07), rs1800947 (0.08), rs1205 (0.35), rs876538 (0.23), and rs11265257 (0.40). In a preliminary analysis, unadjusted for background inflammatory status (ESR), significant associations between serum CRP and SNPs rs1205, rs11265257, and rs1800947 were observed (Tables S1 and S2). In contrast, ESR was not associated with any *CRP* SNP (Table S3).

Table 1 shows the association of *CRP* SNPs with serum CRP level, incorporating ESR as a covariate to adjust for the underlying inflammatory status of that individual. A conservative Bonferroni correction for six tests would require *p*<0.008 to achieve overall significance, hence both rs1205 and rs1125257 are strongly associated with CRP, with the minor allele associated with lower CRP level. We looked for departure from a simple additive model of allele effect but none was seen.

**Table 1:** Single *CRP* SNP effects on acute-phase serum CRP.

SNP	Discovery Cohort (Patient Set 1)			Replication Cohort (Patient Set 2)			Combined Cohorts		
	β (logCRP)	95% CI	*p*-Value	β (logCRP)	95% CI	*p*-Value	β (logCRP)	95% CI	*p*-Value
rs2808632	−0.004	−0.070 to 0.062	0.906	−0.026	−0.098 to 0.045	0.473	−0.017	−0.070 to 0.035	0.522
rs3093059	0.122	0.011 to 0.232	0.031	−0.003	−0.162 to 0.155	0.967	0.047	−0.057 to 0.151	0.372
rs1800947	−0.095	−0.195 to 0.005	0.062	−0.273	−0.406 to −0.140	<0.0005	−0.169	−0.259 to −0.078	<0.0005
rs1205	−0.095	−0.155 to −0.035	0.002	−0.116	−0.188 to −0.045	0.001	−0.109	−0.159 to −0.058	<0.0005
rs876538	0.000	−0.069 to 0.070	0.991	−0.038	−0.118 to 0.043	0.360	−0.026	−0.083 to 0.032	0.382
rs11265257	−0.096	−0.155 to −0.037	0.001	−0.114	−0.185 to −0.044	0.002	−0.106	−0.157 to −0.056	<0.0005

In patient set 2 the median CRP was 5 mg/l (range 0.25–334; 5th, 95th centiles 0.25, 60). 34.9% of CRP measurements were >10 mg/l. The median ESR was 16 mm/h (range 2–106; 5th, 95th centiles 4, 58). Overall, the level of inflammation observed in the patients in set 2 was therefore slightly less than that observed in set 1, suggesting either less severe disease or better disease control; this does not impact on the interpretation of our findings. The SNP minor allele frequencies were rs2808632 (0.34), rs3093059 (0.05), rs1800947 (0.07), rs1205 (0.34), rs876538 (0.24), and rs11265257 (0.41). Initial analysis of patient set 2 confirmed the association of *CRP* genotype with “raw” serum CRP levels, but not with ESR (Tables S1–S3). Table 1 demonstrates the replication in patient set 2 of the significant association of rs1205 and rs11265257 with ESR-adjusted serum CRP. It also shows strong association for rs1800947, a more rare SNP with only borderline association in patient set 1.

Having replicated the initial genetic associations, we performed a combined analysis using pooled data from both cohorts. In addition to the inclusion of a “cohort” term in our combined model (as outlined in the Methods section), we also looked for potential SNP × cohort interactions that might complicate the interpretation of this analysis. None of the key SNP effects we observed differed significantly from one cohort to another. This combined analysis again emphasised the high level of significance attached to our identified SNPs (Table 1).

While Table 1 shows the highly significant associations between SNPs at *CRP* and serum CRP level, it does not provide easily interpretable parameter estimates. In Table 2 we have therefore shown the geometric mean and 95% confidence intervals (CIs) modelled for each genotype (with reference to the major allele homozygotes) at the associated SNPs, in our combined dataset. It should be reiterated that the geometric mean of the log-transformed CRP distribution can be considered equivalent to the median of the untransformed CRP distribution. Since the predicted CRP levels depend on the background inflammatory status (as assessed by ESR) in this model, we have illustrated expected geometric mean CRP at two levels of background inflammation (ESR 40 mm/h and ESR 80 mm/h).

**Table 2:** CRP parameter estimates (geometric mean) from the combined cohorts.

SNP	Genotype	Genotype Frequency	Parameters when ESR = 40		Parameters when ESR = 80	
			Geometric Mean CRP (mg/l)	95% CI	Geometric Mean CRP (mg/l)	95% CI
rs1800947	G G	0.85	26.1	ref	42.2	Ref.
	G C	0.13	17.7	14.4–21.8	28.6	23.2–35.3
	C C	0.01	12.0	7.9–18.2	19.4	12.8–29.5
rs1205	G G	0.43	28.2	Ref.	45.7	Ref.
	G A	0.45	21.9	19.5–24.6	35.5	31.7–40.0
	A A	0.12	17.0	13.5–21.6	27.6	22.0–35.0
rs11265257	G G	0.35	29.2	Ref.	47.6	Ref.
	G A	0.50	22.9	20.4–25.7	37.3	33.1–41.8
	A A	0.15	17.9	14.1–22.6	29.2	23.1–36.7

Considerable evidence, from the basal CRP literature, supports the hypothesis that there are multiple functional SNPs at the *CRP* locus [23]–[25]. We tested this hypothesis directly in the combined dataset by performing an analysis conditioned on the most significant SNPs and were able to demonstrate an independent association from three SNPs entered simultaneously into the regression model (Table S4). It is therefore evident that an analysis of the effect of *CRP* genotype on serum CRP based on individual SNPs alone is likely to markedly underestimate the overall effect size. We therefore performed a haplotype analysis. This approach has the advantage of providing a realistic estimate of the integrated effect of multiple SNPs on acute-phase serum CRP levels, while also only considering those SNP combinations that are actually found in the population and are of direct clinical relevance.

Table 3 demonstrates the inferred haplotype structure and haplotype frequencies in the two patient sets. Table 4 demonstrates the association of *CRP* haplotypes with serum CRP level, with reference to the commonest haplotype, H1, which is set as the baseline. Once again, individual haplotypes were consistently associated with acute-phase serum CRP with high levels of significance in both patient sets individually and in a combined analysis.

**Table 3:** *CRP* haplotype architecture and frequency.

Haplotype	rs11265257	rs876538	rs1205	rs1800947	rs3093059	rs2808632	Frequency (Patient Set 1)	Frequency (Patient Set 2)
H1	G	G	G	G	T	A	0.29	0.26
H2	A	G	A	G	T	A	0.27	0.27
H3	G	A	G	G	T	C	0.22	0.24
H4	A	G	A	C	T	A	0.08	0.07
H5	G	G	G	G	C	A	0.06	0.05

**Table 4:** *CRP* haplotype effect on acute-phase serum CRP.

Haplotype	Discovery Cohort			Replication Cohort			Combined Cohorts		
	β	95% CI	*p*-Value	β	95% CI	*p*-Value	β	95% CI	*p*-Value
H1	Ref.	—	—	Ref.	—	—	Ref.	—	—
H2	−0.053	−0.096 to −0.009	0.018	−0.254	−0.403 to −0.105	0.001	−0.144	−0.209 to −0.079	<0.0005
H3	−0.033	0.080 to −0.014	0.172	−0.250	−0.396 to −0.104	0.001	−0.105	−0.173 to −0.038	0.002
H4	−0.102	−0.166 to −0.038	0.002	−0.557	−0.776 to −0.339	<0.0005	−0.261	−0.359 to −0.162	<0.0005
H5	0.032	−0.038 to 0.101	0.372	−0.106	−0.366 to 0.154	0.423	−0.044	−0.066 to 0.154	0.431

**Figure 1:**
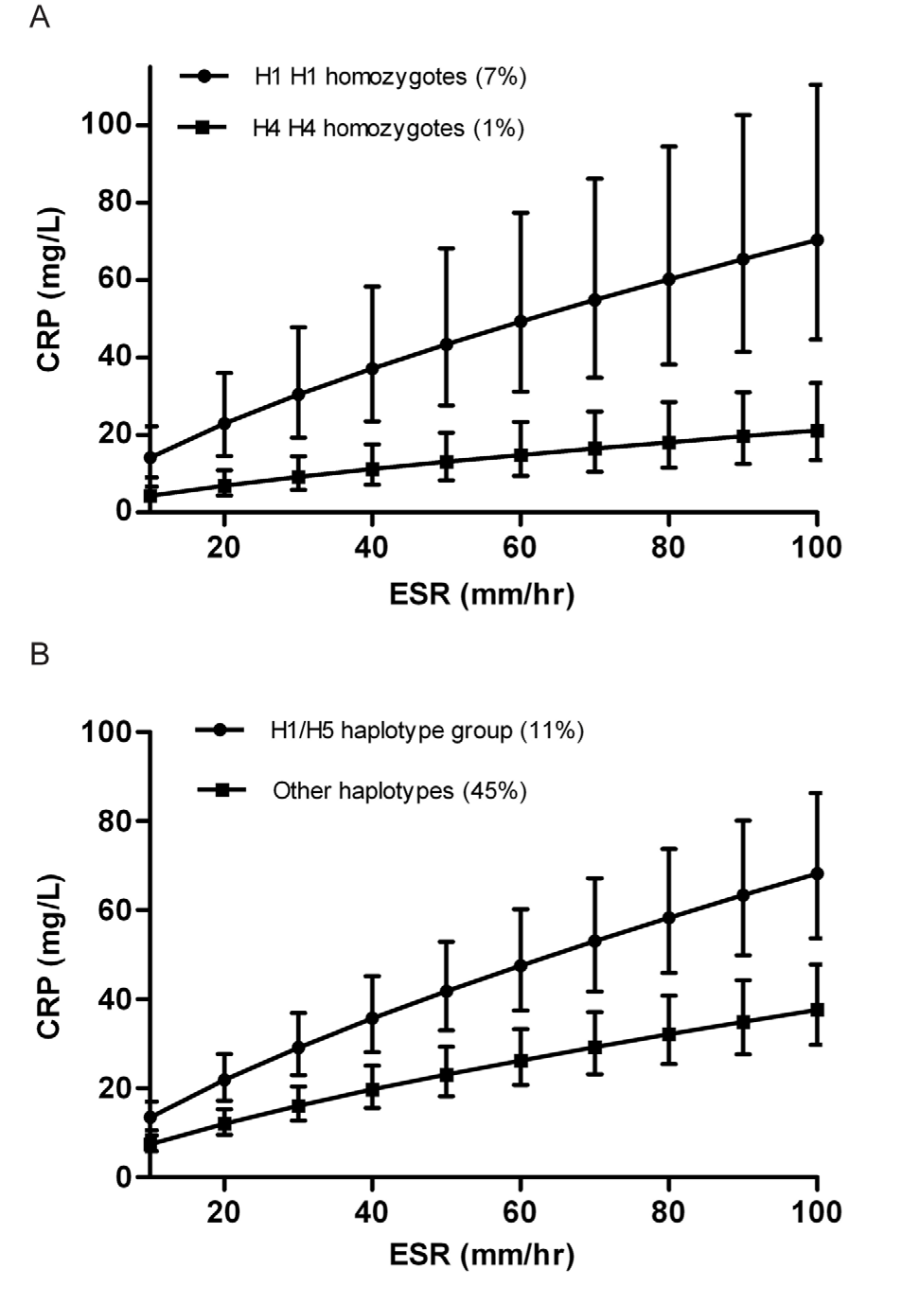
Predicted acute-phase CRP level (95% CI), stratified by haplotype, and according to the ESR-defined level of background inflammation. Annotated by the haplotype pair (A), or grouping (B), represented on the graph, with the population frequency of that combination. Data from the combined cohorts.

Regression parameters were used to illustrate expected CRP values depending on background inflammatory status (defined this by ESR) and *CRP* haplotype. In the setting of chronic inflammation we would expect both ESR and CRP to be at steady state and in equilibrium. Figure 1A illustrates the expected geometric mean (median) CRP (95% CI) for individuals homozygous for haplotype H1 compared with the expected median for H4 homozygotes. H1 homozygotes have a median CRP 232% higher than H4 homozygotes at each level of inflammation (*p*<0.0005).

The percentage of the population carrying any specific haplotype pair is modest (7% for H1 homozygotes and 1% for H4 homozygotes), so we illustrate that the effect on CRP level is not confined solely to rare haplotype pairs but can also be demonstrated between larger groupings in the population. In Figure 1B, we have compared individuals carrying any combination of the H1 or H5 haplotypes (i.e., H1 H1, H1 H5, or H5 H5), with individuals carrying any combination of the remaining haplotypes. The H1/H5 group, constituting 11% of the population have an acute-phase CRP 82% higher than the 45% of individuals carrying two of the remaining haplotypes (*p*<0.0005). Individuals with one haplotype from each of these two groups will naturally have a CRP level falling somewhere in the middle.

We have observed that the magnitude of *CRP* haplotypic effect on serum CRP, over and above the variation in CRP expected due to underlying inflammatory status, is therefore large. To exclude the possibility that these observations were being unduly influenced by rare data outliers we performed additional analyses. Firstly we reran the model after excluding the 5% of individuals with the highest or lowest overall CRP levels. No meaningful changes to the observed significance levels or parameter estimates were observed (Table S5). Secondly we assessed the impact of individuals to the model by their Cook's distances. All Cook's distances were <1, and in addition an exclusion of the 5% of individuals with the greatest Cook's distance again resulted in no material change to the overall effect sizes and significance levels (Table S6).

## Discussion

### Summary and Comparison with Current Literature

We have shown a highly significant association between *CRP* genotype and acute-phase serum CRP level, and to our knowledge we are the first to demonstrate this in a group of patients that includes a considerable number with chronic active inflammation (40% have serum CRP >10 mg/l). We believe that the size of the genetic effect we have observed is large enough to have a clinically relevant impact on the assessment of inflammatory disease activity, which in turn may influence therapeutic decision making.

A key issue in the study of acute-phase CRP genetics is how to quantify the level of inflammatory stimulus driving the acute-phase response. Without this adjustment, random error introduced by varying levels of inflammation across our study population may be so large as to mask the genetic effects owing to variation at the *CRP* locus. We explained in the introduction that we believe that the incorporation of ESR into our analysis enables us to quantify the background level of inflammation in the chronic inflammatory setting. We hypothesized that there is no biological plausibility for a causal link to underlie the normal CRP/ESR correlation. The data from this study provide additional support for this assumption, by showing no association between *CRP* polymorphisms and ESR, and therefore support the hypothesis that CRP and ESR are highly correlated, but truly independent, markers of inflammation.

The use of ESR allowed us to find, with high levels of significance, SNPs and haplotypes associated with high and low acute-phase CRP expression. Existing studies addressing this question have all measured the magnitude of CRP rise following a single noxious stimulus. The largest of these looked at the postmyocardial infarction CRP rise and, like us, found higher levels in carriers of the rare allele of rs3093059 and also the *A* allele of rs3091244, which is on the same haplotype. Conversely lowest CRP increases were seen in carriers of the rare alleles of rs1800947 and rs1205 and the haplotype (H4), which contains both of these SNPs [12]. Other studies have found an association of rs1800947 with lower serum CRP after coronary artery bypass surgery and after oesophagectomy, and rs1130864 with higher serum CRP postcoronary artery bypass surgery [10],[11],[13]. All these studies are complicated by the inability to reliably compare surgical trauma from one procedure to another. Early studies also showed an association between rs1130864 and CRP levels following periodontal therapy and following strenuous exercise, but these stimuli only elicited a low-grade acute-phase response [9],[13]. The findings reported in this study therefore confirm those of earlier reports, but extend them to demonstrate highly significant genetic effects on CRP levels in the setting of chronic inflammation.

In terms of the magnitude of the genetic effect, there is a great deal of variability in the literature discussed above. For example in the cardiac surgery study of Brull and colleagues individuals homozygous for the common allele of rs1130864 had CRP levels that were only 20% higher than heterozygotes or individuals homozygous for the rare allele (164 versus 194 mg/l) [13]. In contrast Suk et al., who examined the increase in CRP concentration following myocardial infarction saw almost 600% difference between genotypes associated with low and high CRP concentrations (11 versus 77 mg/l) [12]. Much of this difference can undoubtedly be explained by the SNP chosen for the study, and also methodological issues such as the level of control for underlying inflammatory stimulus. It is clear, however, that the large effect of *CRP* genotypes that we have observed in this study in the setting of chronic inflammation, is consistent with large effects that have already been described following acute stimuli (such as those described by Suk et al. [12].).

There is also consistency between the SNPs we find associated with elevated CRP in chronic inflammation and those documented in basal CRP studies. In particular both rs1205 and rs1800947 have been widely associated with lower basal CRP expression [2]–[5]. Similar to the acute-phase literature, there is also variation in the magnitude of genotype effects in basal CRP studies, ranging from an approximate 50% difference to a difference of more than 500% in CRP concentrations between high and low *CRP* associated genotypes, from study to study and depending on which SNP or haplotype is examined [1],[2],[5]. It is possible, therefore, that the effect we report in the setting of chronic inflammation is simply an extension of the same effect that is observed with basal CRP. This observation is of interest, because it suggests that the low grade stimulation of CRP expression by “metabolic stress” may share a common pathway with the expression of CRP in response to classical inflammatory stimuli [2],[26].

### Clinical Implications of the Data

By incorporating ESR we can use the model parameters to calculate the expected median CRP for an individual carrying any combination of *CRP* haplotype at any level of background inflammation. This finding showed that the genetic associations translated into marked and clinically relevant effects on acute-phase CRP. Although serum CRP is interpreted as part of a complete clinical picture, we propose that, for a newly symptomatic patient, a CRP of 9 mg/l would be reassuring and might trigger a different clinical decision from a CRP of 30 mg/l; similarly for a patient with known inflammatory disease a CRP of 15 mg/l may be observed, but a CRP of 50 mg/l is likely to trigger a change in treatment; yet our data suggest that these CRP differences could occur simply owing to genetics, independent of any difference in underlying disease activity.

In addition to these intuitive examples, it is worth considering specific clinical algorithms that incorporate CRP. For example, rheumatoid arthritis disease activity is frequently assessed using the DAS-28 score (incorporating a count of tenderness and swelling at 28 specific joints, a global assessment of disease activity by visual analogue scale [VAS], and a biochemical marker of inflammation, either ESR or CRP [http://www.reuma-nijmegen.nl/www.das-score.nl/index.html]). In the United Kingdom a DAS-score threshold is used to determine who receives funding for expensive biological therapies. While the DAS28-ESR is more frequently used at present, it seems inevitable that the DAS28-CRP will increase in popularity, and this is where the effect of genetics becomes important [27]. A patient with a genetically determined CRP of 66 mg/l, six swollen and tender joints, and a VAS of 50 mm will have a DAS score of 5.24. A patient with a genetically determined low acute-phase CRP of 20 mg/l, and an identical VAS would require an additional five tender or ten swollen joints to achieve this DAS score. In other words, a patient with genetically determined low CRP would be required to have considerably worse disease to be considered for the same treatment as a patient with genetically determined high CRP.

A review of the recent literature reveals multiple studies proposing a predictive value for acute-phase CRP; including the prediction of all-cause hospital mortality, death and complications in various critical care scenarios, cancer relapse, and the progression of inflammatory arthritis [28]–[35]. Our data do not contradict these study findings, but they suggest that in some situations the predictive ability of CRP could be significantly improved by the provision of a genetic adjustment. Ironically, while CRP measurement offers many advantages over ESR, we have demonstrated a potential drawback in that common genetic variants strongly influence serum levels. In contrast ESR, which is determined by a number of different physiological processes (as discussed earlier), would seem less likely to be strongly influenced by any individual genetic variant or locus (this, of course, remains to be tested).

Although our study was conducted only in patients of white European ancestry, a comparison with existing studies in different ethnic groups raises the possibility of population differences in the acute-phase CRP response, because of population differences in the haplotype architecture at *CRP*. Of the trans-ethnic mapping studies we are aware of, the most extreme difference appears to be between African American and Filipino populations [36],[37]. In African Americans haplotypes tagged by the minor allele of rs180097 (H4) are very rare (<1%), but in Filipinos this haplotype is common (10%). Similarly haplotypes with the common allele of rs1800974 but the rare allele of rs1205 (H2) are found on 16% of African American but on 43% of Filipino chromosomes. Both these haplotypes are associated with lower acute-phase CRP, and it is intriguing to question whether this would translate into a global tendency towards lower acute-phase CRP in Filipinos compared with African Americans. This hypothesis clearly requires experimental confirmation.

We would also like to speculate on the wider issue of whether the magnitude of acute-phase CRP rise in response to inflammatory stimuli has any implications for patients in terms of the physiological or pathological response to disease. Producing more CRP may offer benefits, perhaps by enhancing clearance of pathogenic bacteria, or by enhancing the clearance of apoptotic cells and reducing susceptibility to systemic autoimmunity [8]. However excessive CRP production may also have deleterious consequences, by promoting inflammation and tissue damage in unwanted situations. This finding has been demonstrated in a rat model of myocardial infarction, where injection of human CRP is associated with a poor outcome; a situation that can be reversed by synthetic CRP inhibitors [23]. Whether naturally occurring, genetically determined variation in the level of acute-phase response translates into differing outcomes in these varied clinical situations remains to be tested.

### Study Limitations

We recognise the limitations to our study. There may be considerable heterogeneity within our study populations, which were recruited over four different sites, with CRP and ESR quantified in four different laboratories. We recognise that although patients self-reported white European ancestry, there may be subtle differences in population ancestry, particularly between patients recruited from the Northern and Southern hemispheres, and that this has not been accounted for by a principle components analysis. In addition we were unable to account for variables such as medication history, which may be important given the reported effect of drugs such as statins on basal CRP levels.

Although our study aim was to assess the influence of genetics on the acute-phase CRP response, we appreciate that 60% of our study participants had a serum CRP concentration of ≤10 mg/l and only 8% had very high serum concentration of CRP (>50 mg/l). The recruitment of patients with uncontrolled inflammation is of course difficult as these patients tend to be aggressively treated.

As this study was conducted in patients with rheumatoid arthritis we need to question whether or not our data can be extrapolated to other chronic inflammatory diseases. *CRP* itself has never been reported as a susceptibility gene in rheumatoid arthritis and, although we did not perform a disease association study here, the allele frequencies we observe in our patient sets are very close to those widely reported elsewhere for non-rheumatoid arthritis patients. We therefore have no reason to suppose that rheumatoid arthritis is a “special case” in terms of its influence on the acute-phase response. We would therefore hypothesize that our findings can be extrapolated to other chronic inflammatory diseases.

### Conclusion

CRP can be measured easily and standard assays offer great accuracy. While the extreme sensitivity and rapid response to changing levels of inflammation make CRP measurement an invaluable clinical tool, our data introduce a note of caution. Acute-phase CRP may be strongly influenced by common genetic variants and CRP concentrations should therefore be interpreted in light of this. If technology advances to the stage of allowing rapid bedside genetic testing, then a personalised, genetically adjusted CRP level may prove to be a useful diagnostic and predictive biomarker.

## Supporting Information

Table S1 SNP associations with "raw" serum CRP (unadjusted for ESR).
(0.03 MB DOC)

Table S2 CRP parameter estimates (geometric mean), unadjusted for ESR.
(0.04 MB DOC)

Table S3 SNP associations with ESR.
(0.03 MB DOC)

Table S4 Conditional CRP-SNP associations.
(0.03 MB DOC)

Table S5 *CRP* haplotype effect on acute-phase serum CRP: Extreme CRP values excluded.
(0.03 MB DOC)

Table S6 *CRP* haplotype effect on acute-phase serum CRP: Top 5% excluded by Cook's D.
(0.03 MB DOC)

## Abbreviations

CI: confidence interval
CRP: C-reactive protein
ESR: erythrocyte sedimentation rate
