# Supplementary material for: A Genetic Association Study of Serum Acute-Phase C-Reactive Protein Levels in Rheumatoid Arthritis: Implications for Clinical Interpretation
Source: PLoS Med. 2010 Sep 21;7(9):e1000341. doi: 10.1371/journal.pmed.1000341 (PMC2943443; doi:10.1371/journal.pmed.1000341)
Supplement: Table S1 — SNP associations with "raw" serum CRP (unadjusted for ESR). (0.03 MB DOC) [file pmed.1000341.s001.doc]

**Table S1: SNP associations with “raw” serum CRP (unadjusted for ESR)**

|  | Discovery Cohort (Patient set 1) | | |  | Replication Cohort (Patient set 2) | | |
| --- | --- | --- | --- | --- | --- | --- | --- |
| SNP | β (logCRP) | 95% CI | P |  | β (logCRP) | 95% CI | P |
| rs2808632 | 0.014 | -0.066, 0.093 | 0.734 |  | 0.000 | -0.091, -0.091 | 0.998 |
| rs3093059 | 0.097 | -0.037, 0.231 | 0.155 |  | 0.066 | -0.268, 0.135 | 0.519 |
| rs1800947 | -0.135 | -0.257, 0.012 | 0.031 |  | -0.186 | -0.357, -0.014 | 0.034 |
| rs1205 | -0.097 | -0.170, -0.024 | 0.010 |  | -0.126 | -0.217, -0.035 | 0.007 |
| rs876538 | -0.009 | -0.093, 0.075 | 0.832 |  | -0.055 | -0.158, 0.048 | 0.297 |
| rs11265257 | -0.087 | -0.159, -0.015 | 0.018 |  | -0.107 | -0.198, -0.016 | 0.021 |
