# Supplementary material for: A Genetic Association Study of Serum Acute-Phase C-Reactive Protein Levels in Rheumatoid Arthritis: Implications for Clinical Interpretation
Source: PLoS Med. 2010 Sep 21;7(9):e1000341. doi: 10.1371/journal.pmed.1000341 (PMC2943443; doi:10.1371/journal.pmed.1000341)
Supplement: Table S2 — CRP parameter estimates (geometric mean), unadjusted for ESR. (0.04 MB DOC) [file pmed.1000341.s002.doc]

**Vyse Table S2: CRP parameter estimates (geometric mean) – unadjusted for ESR**

| SNP | Genotype | Discovery Cohort (Patient set 1) | | |  | Replication Cohort (Patient set 2) | | |
| --- | --- | --- | --- | --- | --- | --- | --- | --- |
|  |  | Genotype frequency | Geometric mean CRP (mg/L) | 95% CI |  | Genotype frequency | Geometric mean CRP (mg/L) | 95% CI |
| rs1800947 | G G | 0.84 | 13.6 | ref |  | 0.88 | 6.3 | ref |
|  | G C | 0.14 | 10.0 | 7.5, 13.2 |  | 0.12 | 4.1 | 2.8, 6.1 |
|  | C C | 0.01 | 7.3 | 4.2, 12.9 |  | 0.01 | 2.7 | 1.2, 5.9 |
| rs1205 | G G | 0.42 | 15.3 | ref |  | 0.45 | 7.1 | ref |
|  | G A | 0.47 | 12.2 | 10.3, 14.5 |  | 0.43 | 5.4 | 4.3, 6.6 |
|  | A A | 0.11 | 9.8 | 8.3, 13.7 |  | 0.12 | 4.0 | 2.6, 6.1 |
| rs11265257 | G G | 0.35 | 15.3 | ref. |  | 0.34 | 7.2 | ref |
|  | G A | 0.51 | 12.6 | 10.6, 14.8 |  | 0.50 | 5.6 | 4.6, 6.9 |
|  | A A | 0.14 | 10.3 | 7.4, 14.3 |  | 0.16 | 4.4 | 2.9, 6.7 |
