# Supplementary material for: A Genetic Association Study of Serum Acute-Phase C-Reactive Protein Levels in Rheumatoid Arthritis: Implications for Clinical Interpretation
Source: PLoS Med. 2010 Sep 21;7(9):e1000341. doi: 10.1371/journal.pmed.1000341 (PMC2943443; doi:10.1371/journal.pmed.1000341)
Supplement: Table S3 — SNP associations with ESR. (0.03 MB DOC) [file pmed.1000341.s003.doc]

**Table S3: SNP associations with ESR**

|  | Discovery Cohort (Patient set 1) | | |  | Replication Cohort (Patient set 2) | | |
| --- | --- | --- | --- | --- | --- | --- | --- |
| SNP | β (logESR) | 95% CI | P |  | β (logESR) | 95% CI | P |
| rs2808632 | 0.025 | -0.052, 0.102 | 0.524 |  | 0.025 | -0.025, 0.076 | 0.325 |
| rs3093059 | -0.005 | -0.135, 0.125 | 0.942 |  | -0.053 | -0.165, 0.058 | 0.349 |
| rs1800947 | -0.072 | -0.192, 0.049 | 0.243 |  | 0.053 | -0.043, -0.149 | 0.280 |
| rs1205 | -0.006 | -0.077, -0.066 | 0.872 |  | -0.012 | -0.062, 0.039 | 0.654 |
| rs876538 | -0.004 | -0.085, 0.077 | 0.919 |  | -0.013 | -0.070, 0.044 | 0.650 |
| rs11265257 | 0.003 | -0.067, 0.074 | 0.924 |  | 0.008 | -0.043, 0.058 | 0.758 |
