# Supplementary material for: A Genetic Association Study of Serum Acute-Phase C-Reactive Protein Levels in Rheumatoid Arthritis: Implications for Clinical Interpretation
Source: PLoS Med. 2010 Sep 21;7(9):e1000341. doi: 10.1371/journal.pmed.1000341 (PMC2943443; doi:10.1371/journal.pmed.1000341)
Supplement: Table S4 — Conditional CRP-SNP associations. (0.03 MB DOC) [file pmed.1000341.s004.doc]

**Table S4A**: Conditional *CRP* SNP associations*

| SNP | β (logCRP) | 95% CI | P |
| --- | --- | --- | --- |
| rs1205 | -0.130 | -0.191, -0.068 | <0.0005 |
| rs2808632 | -0.090 | -0.150, -0.030 | 0.003 |
| rs1800947 | -0.114 | -0.210, -0.018 | 0.020 |

*SNPs entered into model simultaneously

**Table S4B**: Conditional *CRP* SNP associations* - Alternative model

| SNP | β (logCRP) | 95% CI | P |
| --- | --- | --- | --- |
| rs1125257 | -0.100 | -0.156, -0.045 | <0.0005 |
| rs1800947 | -0.129 | -0.224, -0.033 | 0.008 |
| rs2808632 | -0.058 | -0.113, -0.003 | 0.040 |

*SNPs entered into model simultaneously
