# Supplementary material for: A Genetic Association Study of Serum Acute-Phase C-Reactive Protein Levels in Rheumatoid Arthritis: Implications for Clinical Interpretation
Source: PLoS Med. 2010 Sep 21;7(9):e1000341. doi: 10.1371/journal.pmed.1000341 (PMC2943443; doi:10.1371/journal.pmed.1000341)
Supplement: Table S5 — CRP haplotype effect on acute-phase serum CRP: Extreme CRP values excluded. (0.03 MB DOC) [file pmed.1000341.s005.doc]

**Table S5A**: *CRP* haplotype effect on acute-phase serum CRP – Top 5% excluded by CRP.

|  | Combined Cohorts | | |
| --- | --- | --- | --- |
| Haplotype | β (logCRP) | 95% CI | P |
| H1 | Ref |  |  |
| H2 | -0.142 | -0.206, -0.078 | <0.0005 |
| H3 | -0.119 | -0.185, -0.052 | <0.0005 |
| H4 | -0.250 | -0.347, -0.154 | <0.0005 |
| H5 | -0.065 | -0.174, 0.043 | 0.238 |

**Table S5B**: *CRP* haplotype effect on acute-phase serum CRP – Bottom 5% excluded by CRP.

|  | Combined Cohorts | | |
| --- | --- | --- | --- |
| Haplotype | β (logCRP) | 95% CI | P |
| H1 | Ref |  |  |
| H2 | -0.118 | -0.181, -0.055 | <0.0005 |
| H3 | -0.077 | -0.143, -0.011 | 0.022 |
| H4 | -0.243 | -0.338, -0.147 | <0.0005 |
| H5 | -0.038 | -0.144, 0.068 | 0.482 |
