# Supplementary material for: A Genetic Association Study of Serum Acute-Phase C-Reactive Protein Levels in Rheumatoid Arthritis: Implications for Clinical Interpretation
Source: PLoS Med. 2010 Sep 21;7(9):e1000341. doi: 10.1371/journal.pmed.1000341 (PMC2943443; doi:10.1371/journal.pmed.1000341)
Supplement: Table S6 — CRP haplotype effect on acute-phase serum CRP: Top 5% excluded by Cook's D. (0.03 MB DOC) [file pmed.1000341.s006.doc]

**Table S6: *CRP* haplotype effect on acute-phase serum CRP – Top 5% excluded by Cook’s D.**

|  | Combined Cohorts | | |
| --- | --- | --- | --- |
| Haplotype | β (logCRP) | 95% CI | P |
| H1 | Ref |  |  |
| H2 | -0.150 | -0.210, -0.090 | <0.0005 |
| H3 | -0.136 | -0.198, -0.073 | <0.0005 |
| H4 | -0.253 | -0.349, -0.158 | <0.0005 |
| H5 | -0.046 | -0.148, 0.056 | 0.378 |
